# MF–NF Treatment Train for Pig Manure: Nutrient Recovery and Reuse of Product Water

**DOI:** 10.3390/membranes12020165

**Published:** 2022-01-30

**Authors:** Prantik Samanta, Hannah Marie Schönettin, Harald Horn, Florencia Saravia

**Affiliations:** 1 DVGW-Research Center, Water Chemistry and Water Technology, Engler-Bunte-Ring 9a, 76131 Karlsruhe, Germany; harald.horn@kit.edu (H.H.); saravia@dvgw-ebi.de (F.S.); 2 Engler-Bunte-Institut, Water Chemistry and Water Technology, Karlsruhe Institute of Technology, Engler-Bunte-Ring 9a, 76131 Karlsruhe, Germany; hannah.schoen@gmx.de

**Keywords:** pig manure, MF–NF train, resource recovery, volume reduction, water reuse

## Abstract

The livestock industry negatively impacts the environment by producing high organic and mineral loaded manure and wastewater. On the contrary, manure is also considered as the major focal point of resource recovery. The microfiltration (MF) process in manure treatment is well known for being the least complex and highly energy efficient. However, the major fraction of the dissolve nutrients easily bypasses the MF membranes. In this research work, we reported the efficiency of using MF–nanofiltration (NF) treatment train in a dead-end filtration system for the treatment of raw manure. The objectives were to produce nutrient rich separate streams in reduced volumes and a particle and pathogen-free product water. MF removed TSS above 98% and the COD and phosphorus (P) retention were noticed above 60 and 80%, respectively, within a reduced MF concentrate volume, which accounted for 40% of the initial feed volume. The NF of MF permeate by NF270 showed most promising results by concentrating overall 50 and 70% of the total nitrogen (TN) and potassium (K) within a reduced NF concentrate volume, which accounted for 30% of the initial MF feed volume. Finally, the MF–NF treatment train of raw pig manure could produce a particle-free product water that can be reused in farms to wash barns, to irrigate nearby cultures, or can be applied to specific fields based on the demand.

## 1. Introduction

The livestock industry negatively impacts the environment by producing high organic and mineral loaded manure and wastewater [1,2]. Manure contributes to environmental pollution by releasing ammonia and nitrous oxide into the atmosphere [3], by leaching nitrate mainly into ground water [4], and by increasing the soil acidification as well [5]. Excess nitrogen (N) and phosphorus (P) that are released due to manure application degrades the overall aquatic ecosystem [6]. This forced the European Community to implement nitrate directive guidelines to control the groundwater nitrate pollution [7].

On the contrary, manure is highly popular in agricultural applications for containing plant essential nutrients [8], in biopolymer production due to substantial volatile fatty acids concentrations [9], and most importantly in biogas production for energy recovery as well [10]. Therefore, the nutrient recovery techniques from manure are in high demand, despite the presence of high solid contents, organic materials, and its potential hazardous properties [11].

The previously mentioned nutrient recovery processes from manure, such as hydrogel application [12], calcium phosphate precipitation [13], ammonia stripping [14], and struvite precipitation [15] have proven to be very complex and required high chemical and energy inputs. Additionally, the drawbacks of using conventional mechanical processes to treat manure such as sedimentation, centrifugation, and pressurized filtrations have been well described previously [16]. Mechanical processes such as sedimentation and centrifugation retained up to 56 and 44% of dry matter of manure. Whereas, MF could retain, on average, 75% of the dry matter content of manure. Consequently, the total phosphorus retention by MF was found 30–50% higher than the sedimentation and the centrifugation processes. However, the total nitrogen retention did not show many differences due to its significant presence in the liquid part of the manure [16,17,18]. Therefore, using membrane separation processes as an alternative provide an edge to the above-mentioned techniques in producing particle and pathogen-free, nutrient rich separate streams with relatively lower maintenance and operating costs [19].

MF can retain particles that range between 0.1 and 10 µm. Hence, it is well suited to retaining nutrients like P, which are mostly related to the solid phase in manure [17]. However, the dissolved nutrients such as K and N (mainly present as NH_4_^+^-N) mostly pass through the MF membranes [11]. NF is capable of retaining major parts of the total N and K within a smaller concentrate volume [16,17]. Therefore, the released nutrients can be concentrated using nanofiltration as a second step after MF. NF is also well known for its high micropollutants (e.g., antibiotics, antibiotics resistance genes, etc.) removal capacity [20], which enables the final product water to be reused to wash barns, irrigate nearby cultures, or apply on fields based on demand [17].

Limited studies have been reported on the application of NF as a post-treatment process after MF for manure and digestate treatment so far. However, none of the studies have compared between loose and tight NF membranes and commented on their efficiencies as a post-treatment process after MF. Therefore, the objectives of using an MF–NF treatment train in this research study to treat raw manure are to (i) perform solid–liquid separation by MF to produce nutrient rich separate streams in reduced volumes, (ii) to further concentrate the dissolved nutrients from MF permeate using different NF membranes and compare their efficiencies, and finally (iii) to produce a particle and pathogen-free product water.

## 2. Materials and Methods

### 2.1. Pig Manure Sampling

Pig manure samples from pits of sampling sites 1 and 2, located in the state of Baden Württemberg, Germany, were collected in June 2020. Raw pig manure from the pit of sampling site 3, located in the state of lower Saxony, Germany, was collected in April 2020. The samples were collected in 10–30 L canisters and quickly stored at 4 °C in the dark [21] for further experiments. Site 1 contained over 500 pigs, whereas sites 2 and 3 were smaller farms. They contained overall 150–200 pigs each. The pigs of sites 2 and 3 were several months younger than the pigs of site 3. In addition, their diverse location and the growing culture resulted in different manure qualities.

### 2.2. Membrane Characteristics

The raw manure samples were initially filtered by using 0.45 µm pore sized MF membranes to eliminate the suspended solids. The MF membrane characteristics are mentioned in a previous research study by Wei, Laborie, Aim, and Amy [22]. Three different loose NF membranes, NF270, HC50, and NTR7450 were further used for post-treatment of the MF permeate. The NF membrane characteristics are listed in Table 1.

### 2.3. Membrane Filtration Processes

#### 2.3.1. Microfiltration

Manure samples were initially sieved through a 1 mm sieve to eliminate the particles (≥1 mm). The samples were then prefiltered in a dead-end stirred cell membrane filtration system, manufactured by Merck KGaA Germany (Supporting Information Figure S1), by using 0.45 µm pore sized MF membranes. The internal membrane diameter was 14 cm and the effective membrane area was calculated to be 154 cm^2^ in the filtration cell. An initial feed volume of 500 mL was introduced in the feed tank for MF experiments. The filtrations were then performed by applying 1 bar pressure (N_2_ gas, air liquid) and the rotational speed was maintained at 400 rpm. Consequently, 300 mL of permeate were collected in a sterile vial. The filtration was repeated twice for each manure sample to collect a total of 600 mL of MF permeate. The temperature was 25 °C ± 1 during the prefiltration experiments. The MF permeate samples were further analyzed for retention calculation.

#### 2.3.2. Nanofiltration

An MF permeate volume of 200 mL each was used as the feed volume for the following NF experiments which were done using the same stirred cell dead-end filtration set up as mentioned in Section 2.3.1. Consequently, 100 mL of permeate were collected in a sterile vial after each NF experiment. The NF experiments were performed at 6.5 bar as the system could sustain maximum of 7 bar pressure. The rest of the filtration conditions were kept the same as the MF experiments. Similarly, pure water (Merck Millipore, Darmstadt, Germany) permeability (PWP) was measured at 6.5 bar pressure before and after each NF experiment. The NF permeate samples were further analyzed for retention calculation.

### 2.4. Analytical Process

Total suspended solids (TSS) and volatile suspended solids (VSS) were measured according to the established methods [27]. The total chemical oxygen demand (tCOD), ammonium nitrogen (NH_4_^+^-N), and total phosphorus (Tot-P) were measured using LCK014, LCK304, and LCK349 quick test kits (Hach Lange GMBH, Düsseldorf, Germany), respectively. The dissolved organic carbon (DOC) and dissolved total nitrogen (DTN) were measured with a TOC analyzer (Shimadzu TOC-V CPN, Kyoto, Japan). Organic acid anion concentrations were measured by an ion chromatography (IC) system (790 Personal Metrohm, Herisau, Switzerland). The cations were measured by inductively coupled plasma–optical emission spectrometry (ICP-OES, VistaPRO CCD, Fa. Varian, Mulgrave, Australia). Electrical conductivity and pH were measured by a portable multimeter (WTW Multi 350i, Xylem, Weilheim, Germany).

### 2.5. Calculated Parameters

The permeate flux (*J*) was determined by the ratio of the permeate flow rate (*Q_p_*) to effective membrane area (*A_m_*) [18]:

(1) $$J=\left(\frac{Q_{p}}{A_{m}}\right)\;(L\;m^{-2}\;h^{-1})$$

The pure water permeability (*PWP*) was calculated by the ratio of the pure water flux (*J_w_*) to the applied pressure (*TMP*):

(2) $$PWP=\left(\frac{J_{w}}{TMP}\right)\;(L\;m^{-2}\;h^{-1}{\mathrm{bar}}^{-1})$$

The retention calculation was performed by following equation [18]:

(3) $$R=\left(1-\left(\frac{C_{p}}{C_{f}}\right)\right)\times 100\;\left(\%\right)$$

where, *R* is the calculated retention in percent (%). *C_p_* and *C_f_* are the permeate and feed concentration of any parameter at a given recovery.

The permeate volume recovery was calculated according to equation [18]:

(4) $$Rec=\left(1-\left(\frac{V_{p}}{V_{f}}\right)\right)\times 100\;\left(\%\right)$$

where, *Rec* is the calculated recovery in percent (%). *V_p_* and *V_f_* are the permeate and feed volume at a given time.

## 3. Results and Discussion

### 3.1. Chemical Characterization of Manure

Pig manure characteristics vary strongly depending on various parameters such as pig feed, manure storage conditions (site location, storage duration, temperature, etc.), and manure sampling methods [28]. Chemical characteristics of pig manure slurry of three investigated sites are given in Table 2. A standard deviation of maximum 5% within the measured values was observed.

In general, TSS is the combination of suspended solids that can be degraded by microorganisms and non-degradable suspended solids. The TSS ranged within 3–5 gL^−1^ in all three manure samples. Similar results were shown in previous literatures for raw manure [29,30]. TSS is also considered as one main contributor of tCOD. This could be noticed in the close ratios between tCOD and TSS among the manure samples [31]. Relatively lower NH_4_^+^-N concentration was found in a sample of site 2. This could be attributed to various facts such as (i) conversion of ammonium to ammonia due to longer storage may lead to further valorization or evaporation [32] and (ii) different pig feed and growth stage as well [33]. The Tot-P concentration was lowest in the sample of site 2, followed by site 1 and site 3. Only 19% of the Tot-P was dissolved in the sample of site 2. The number was raised to 55 and 44% for the samples of sites 1 and 3, respectively. This reflects the dominant presence of P in the solid fraction of manure.

The DTN in pig manure is the sum of dissolved organic N, dissolved NH_4_^+^-N, and dissolved nitrate N, although nitrate is not typically present in manure [34]. However, parts of the organic N convert into NH_4_^+^-N, which then further converts into ammonia and contributes into total gradual loss of DTN [32]. This might lead to the lowest DTN value for sampling site 2 as the manure storage timing was the longest. Similar DTN values in pig manure samples were mentioned previously [35]. DOC is generally metabolized through volatile fatty acid intermediates [36]. Hence, the acetic acid and DOC concentrations were strongly correlated in manure samples. It was found that the organic carbon from acetic acid made up 44, 30, and 24% of the DOC in the manure samples of sites 1, 2, and 3, respectively.

### 3.2. Microfiltration of Manure

**Figure 1:**
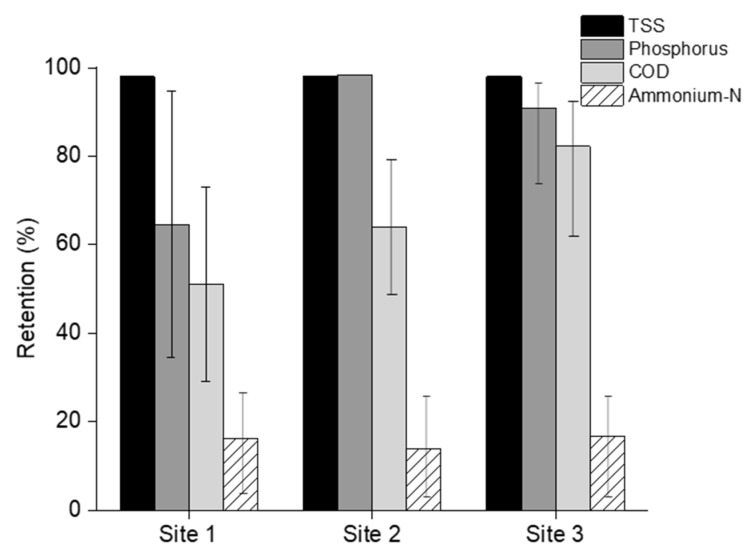
Polymeric MF retention at 60% recovery. Pressure: 1 bar; stirring rate: 400 rpm; temperature: 25 °C.

MF was performed to achieve the solid–liquid separation. Feed samples were collected before the start of each MF experiment. Permeate and concentrate samples were collected after 60% recovery was achieved. The recovery was calculated by following Equation (4). The samples were then analyzed to probe the TSS, Tot-P, COD, and NH_4_^+^-N retention by using Equation (3). The MF retention results are displayed in Figure 1.

**Figure 2:**
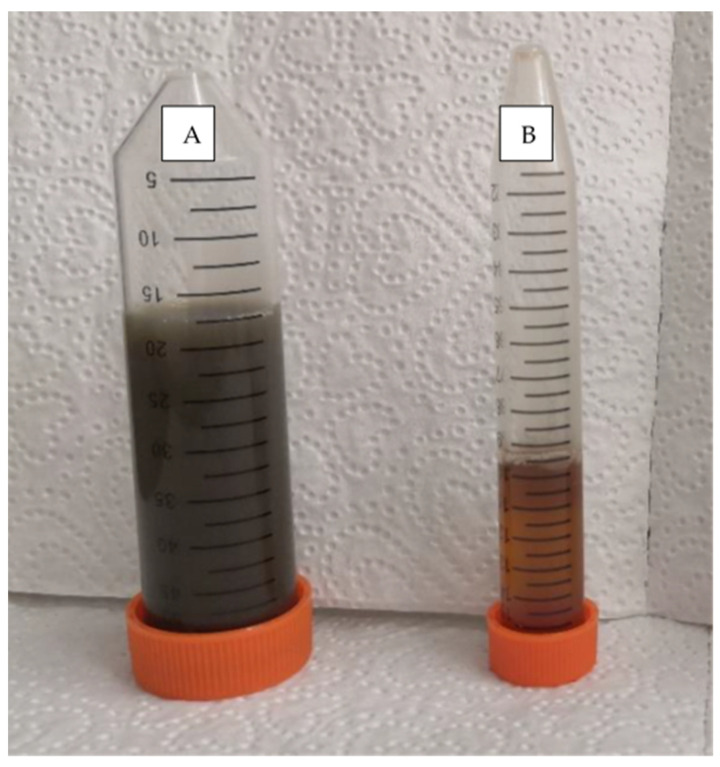
(**A**) MF feed and (**B**) MF permeate after microfiltration performance of site 2 manure.

TSS retention of manure from all three sampling sites was above 98% (Figure 1). TSS-free MF permeate could visibly be observed as a transparent liquid compared to the MF feed (Figure 2). The Tot-P retention remained above 80%. Short filtration duration may lead to retained dissolved P as well. The COD retention of 80% was highest in site 3. In the other sites, the retention remained within 50–60%. Low NH_4_^+^-N retention was found in all sampling sites. The overall NH_4_^+^-N retention stayed within 15–20%.

The efficiency of MF on TSS, colloids, and bacteria removal from mixed liquor and the effluents from biological reactors treating manure is well known [37,38]. Different studies also showed the complete removal of TSS from manure by using polymeric MF as well [39].

Higher retention of Tot-P could be attributed to its linkage with the particles between 0.45 and 10 µm in pig manure [40]. Consequently, Christensen et al. (2009), quantified that more than 70% of the Tot-P in pig manure slurry is associated with particles or colloids [41]. Therefore, it can be stated that the high TSS retention by MF enhanced the Tot-P retention as well. tCOD retention of manure by MF is closely associated with the retention of the particulate organic matter content [39,42]. Hence, the manure sample of site 1 with the lowest TSS content (Table 2) presented the lowest tCOD retention by MF. Similarly, the tCOD retention of site 3 manure, which contains 56% higher TSS than site 1, resulted in 30% higher tCOD retention by MF as well. Previous studies have mentioned the dominant (>88% of TN) presence of NH_4_^+^-N in the liquid fraction of pig manure [41,43]. Consequently, 5–10% mineralization of the organic N during manure storage also supports the above findings [44,45]. Hence, the 15–20% retention of NH_4_^+^-N in the present study can be associated with the mineralized fraction retention by MF. In addition, MF was also expected to remove antibiotic resistance bacteria and pathogens from raw manure as well [46,47].

### 3.3. Nanofiltration of MF Permeate

The objectives of performing the NF of MF permeate were to concentrate the dissolved nutrients (e.g., NH_4_^+^-N, K, and P) and to produce a permeate stream that can be reused. The permeate and concentrate samples were collected after 50% recovery was achieved. The recovery was calculated by following Equation (4). The samples were analyzed to calculate the retention by following Equation (3).

#### 3.3.1. DTN and NH_4_^+^-N Retention

**Figure 3:**
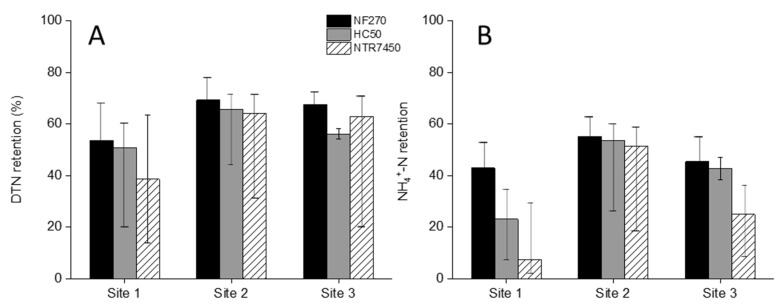
(**A**) DTN and (**B**) NH_4_^+^-N retention of MF permeate at 50% recovery by NF270, HC50, and NTR7450 membranes from all sampling sites. Pressure: 6.5 bar; stirring rate: 400 rpm; temperature: 25 °C.

DTN and NH_4_^+^-N retention by NF membranes are shown in Figure 3. The DTN retention of site 1 manure remained within a range of 40–50% by all NF membranes. However, the retention went above 60% for the other two sites. Interestingly, the DTN feed concentration of site 1 was 15 and 30% higher than that of sites 2 and 3, respectively. The individual membranes presented similar retention trends in all sampling sites. The retention by NF270 was the highest, followed by HC50 and NTR7450 membranes, respectively.

The NH_4_^+^-N contributed approximately 60–70% to the DTN. The overall NH_4_^+^-N retention was nearly 10–20% lower than DTN retention in all sampling sites. The retention trend was mainly unchanged. The NH_4_^+^-N retention differences among the membranes for site 2 remained within 5% only. However, the differences between NH_4_^+^-N retention by NF270 and NTR7450 membranes was found as high as 30 and 15% in sites 1 and 3, respectively. This can be attributed to their respective surface charges and pore sizes as well.

Pig manure can be viewed as a mixed salt solution (Table 2). Therefore, the NH_4_^+^-N retention is assumed to be affected by the retention of the other competing ions as well (Supporting Information, Figure S2). A previous study showed that the increase in competing ions concentration in the feed can reduce the examined ion retention [48]. The NH_4_^+^-N retention by NF membranes is mainly influenced by charge interaction. It was already found that at pH7, NF270 retained 87.5% NH_4_^+^-N, when the filtration was performed under critical pressure. The study stressed the coupling of positively charged NH_4_^+^-N ions with the negatively charged active layer of the membrane [49]. Whereas, filtering dairy manure digested by NF270 resulted in 30–36% NH_4_^+^-N retention [11].

In addition, Hurtado and Cancino-Madariaga [50] also observed that higher NH_4_^+^-N feed concentration resulted in lower retention by NF membranes. It is assumed that the increased feed concentration enhanced NH_4_^+^-N flux through negatively charged NF membranes [51] due to the reduction in the Donnan effect and the neutralization of the membrane surface [52].

#### 3.3.2. Phosphorus and Potassium Retention

**Figure 4:**
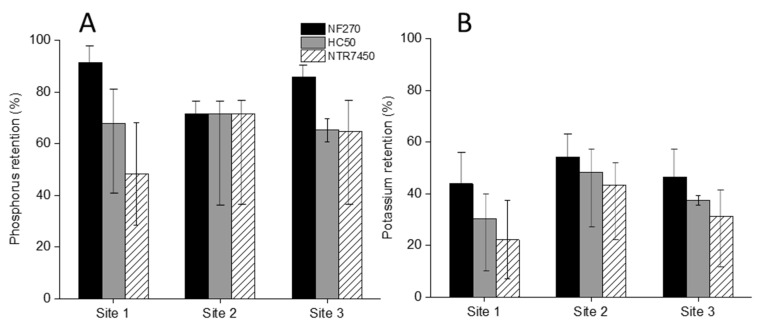
(**A**) Phosphorus and (**B**) potassium retention of MF permeate at 50% recovery by NF270, HC50, and NTR7450 membranes from all sampling sites. Pressure: 6.5 bar; stirring rate: 400 rpm; temperature: 25 °C.

The P retention by NF membranes is presented in Figure 4A. The substantial amount of P retention by MF resulted in a low P feed concentration for NF. The overall P retention was at or above 70%. In particular, NF270 retained above 90 and 80% of the remaining P from manure samples of sites 1 and 3, respectively. These were 10–20% higher than the retention by HC50 and NTR7450 membranes. Interestingly, no such retention differences between the NF membranes were noticed in the case of manure from site 2. Low feed P concentration and even lower permeate P concentration, led the analyzer to yield the limit of quantification as the permeate concentration in this case.

**Figure 5:**
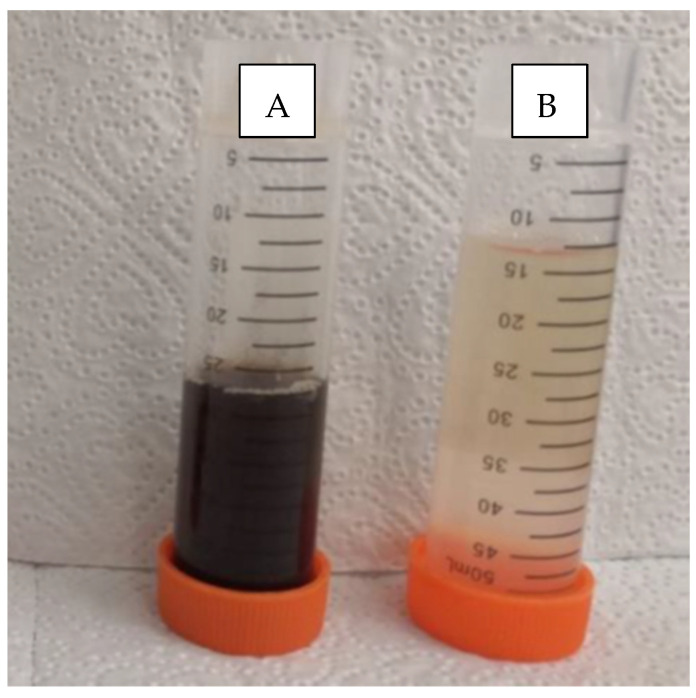
(**A**) NF270 concentrate and (**B**) NF270 permeate of site 2 manure.

The potassium retention by NF membranes is displayed in Figure 4B. No real correlation between the initial feed potassium concentration and the retention was observed. The overall potassium retention was found to be within 22–54%. A similar retention trend was noticed in all manure samples. The highest retention was achieved by NF270. The retention by NTR7450 was 15–20% lower than NF270 and 5–10% lower than HC50 as well. High organic matter retention by NF (Supporting Information, Figure S3) could visibly be noticed (Figure 5) as well.

Previous literature reported 96.4–97.2% P retention, when filtering dairy manure digestate using NF270. They stressed the charge repulsion effect as the main retention mechanism [53]. It is a well-known fact that the dissolved fraction of P in manure is mostly found in the orthophosphate (PO_4_^−3^) form [41]. Ballet, Hafiane, and Dhahbi [54] also reported 99% retention of the divalent (HPO_4_^−2^) form of phosphate by NF NF200 membrane. However, they carried out the experiments in a single salt solution condition, which might show very high retention [48]. Therefore, the higher retention of the trivalent form of dissolved phosphate by negatively charged NF membranes justifies the current findings. Previous literature has reported the proportional relation between the potassium chloride (KCl) feed concentration and the corresponding retention by NF membranes. They reported the range of KCl retention to be between 25 and 45% while filtering KCl solution of 5–15 gL^−1^ feed concentration, using an NF270 membrane [55]. Probably, due to the higher complexity and the presence of different ions and compounds of the feed manure, the trend was not clearly observed in the present study. Masse et al. (2007) noticed that, at higher recovery, the K retention was slightly decreased when filtering pretreated pig manure, with reverse osmosis membranes [17]. Lastly, it is proven that NF retains extracellular antibiotic resistance genes from manure and digestate above 99.99% [56,57]. This facilitated the pathogen-free product water production as well.

### 3.4. Membrane Performance

**Figure 6:**
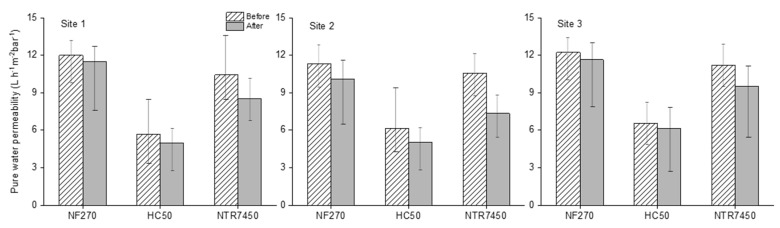
Pure water permeability of NF270, HC50, and NTR7450 membranes, before and after filtering MF permeates from all of the sampling sites. Pressure: 6.5 bar; stirring rate: 400 rpm; temperature: 25 °C.

The permeate flux (J) and pure water permeability (PWP) were calculated by following Equations (1) and (2), respectively. The PWP results of all three NF membranes are displayed in Figure 6. The PWP declined by around 3 L m^−2^ h^−1^bar^−1^ for NTR7450. The permeability decline was reduced to 1–2 L m^−2^ h^−1^bar^−1^ for HC50 and was found to be lowest (0.5–1 L m^−2^ h^−1^bar^−1^) for NF270. However, the stabilized normalized flux (Supporting Information, Equation (S1)) for HC50 and NTR7450 was 0.6–0.7 and 0.5–0.6 L m^−2^ h^−1^, respectively. NF270 presented the lowest normalized flux of 0.2–0.3 L m^−2^ h^−1^ (Supporting Information, Figure S4).

The drop in normalized flux was mainly caused due to the combined effect of reversible and irreversible fouling but the PWP decline after the filtration experiments was associated principally with the irreversible fouling [58]. Since NF270 showed the least fouling, the low normalized fluxes in the experiments with these membranes are presumably associated with the concentration polarization (CP). Winter et al. (2017) noticed that higher CP played an important role for lower MWCO membranes, while filtering natural organic matter [59]. Hence, CP might largely affect the NF270 fluxes while the effect was smallest for the lower rejection NTR7450 membranes. It is also evident that the NTR7450 showed the highest fouling, followed by HC50 membranes.

## 4. Conclusions

The main benefits of the raw manure treatment by a cascade of MF and NF are to produce nutrient rich separate streams in reduced volumes. MF can separate the particulate material and NF can further concentrate the dissolve nutrients. Finally, a particle and pathogen-free product water is generated, which can be further reused in farms. Hence, the overall advantages of using a MF–NF treatment train for raw manure treatment is given below:

i. MF retained phosphorus above 80% within a smaller MF concentrate volume, which accounted for 40% of the initial feed volume. Additionally, the MF permeate contained above 80% of the total nitrogen and most of the dissolve potassium.
ii. NF of the MF permeate by three different NF membranes showed a maximum of 50–70% potassium and NH_4_^+^-N retention, respectively, within smaller NF concentrate volumes, which accounted for 30% of the initial feed volume of MF. Among all of the NF membranes, NF270 showed the most promising retention and was found to be the least prone to fouling.
iii. Finally, the MF–NF treatment train was able to produce a particle-free final product water, which accounted for 30% of the initial feed volume of MF. This has the potential to be reused in farms to wash barns, to irrigate nearby cultures, or can be applied to specific fields based on the demand.

## Figures and Tables

**Table 1:** NF membrane characteristics.

NF Membrane	Supplier	Surface Layer	pH Tolerance	MWCO *”	Water Permeability
				[Da]	[L h^−1^ m^−2^bar^−1^]
NF270	DuPont	Polyamide	3 to 11	300 [23]	13.5 [23]
HC50	Nitto	SPES *	3 to 11	1000 [24]	7.5 [24]
NTR7450	Nitto-Denko	SPES *	3 to 11	2000–3000 [25,26]	9.2 [23]

* sulfonated polyethersulfone (SPES); *” molecular weight cut off (MWCO).

**Table 2:** Pig manure characteristics.

Parameters	Site 1	Site 2	Site 3
TSS (g L^−1^)	3	4.9	4.7
VSS (% of TSS)	83	78	78
tCOD (g L^−1^)	11.3	11.8	10.7
NH_4_^+^-N (g L^−1^)	4.4	2.9	4.4
Tot-P (mg L^−1^)	185	103	283
pH	7.8	7.8	7.9
DOC (g L^−1^)	3.3	3	2.2
DTN (g L^−1^)	3	2	2.5
Dissolved-P (mg L^−1^)	103	19.2	105
Chloride (mg L^−1^)	1745	1083	1674
Sulphate (mg L^−1^)	39	86	108
Acetic acid (mg L^−1^)	3637	2211	1317
Calcium (mg L^−1^)	156	138	175
K (mg L^−1^)	1793	1697	2737
Sodium (mg L^−1^)	895	409	850

## Data Availability

The data that support the findings of this study are available on request from the corresponding author.

## Supplementary Materials

The following Supporting Information can be downloaded at: https://www.mdpi.com/article/10.3390/membranes12020165/s1, Figure S1: Stirred cell dead-end membrane filtration system; Figure S2: (A) COD and (B) DOC retention of MF permeate at 50% recovery by NF270, HC50, and NTR7450 membranes from all sampling sites. Pressure: 6.5 bar; stirring rate: 400 rpm; temperature: 25 °C; Figure S3: Ion retention of MF permeate at 50% recovery by NF270, HC50, and NTR7450 membranes from all sampling sites. Pressure: 6.5 bar; stirring rate: 400 rpm; temperature: 25 °C; Figure S4: Flux during filtrations of NF270, HC50, and NTR7450 membranes while filtering pig manure from all sampling sites. Pressure: 6.5 bar; stirring rate: 400 rpm; temperature: 25 °C; Equation (S1): Normalized flux calculation.
