# Supplementary material for: MF–NF Treatment Train for Pig Manure: Nutrient Recovery and Reuse of Product Water
Source: Membranes (Basel). 2022 Jan 30;12(2):165. doi: 10.3390/membranes12020165 (PMC8875562; doi:10.3390/membranes12020165)
Supplement: Supplementary file 1 [file membranes-12-00165-s001.zip › membranes-1566439-supplementary.pdf]

Supporting information

MF-NF treatment train for pig manure: Nutrient recovery and reuse of product water

Prantik Samanta[a,b], Hannah Marie Schönettin[b], Harald Horn[a,b], Florencia Saravia[a]

[a] DVGW-Research Center at the Engler-Bunte-Institut, Water Chemistry and Water Technology, Karlsruhe Institute of Technology, Engler-Bunte-Ring 9, 76131, Karlsruhe, Germany

[b] Karlsruhe Institute of Technology, Engler-Bunte-Institut, Water Chemistry and Water Technology, Engler-Bunte-Ring 9, 76131, Karlsruhe, Germany

1. Membrane filtration system

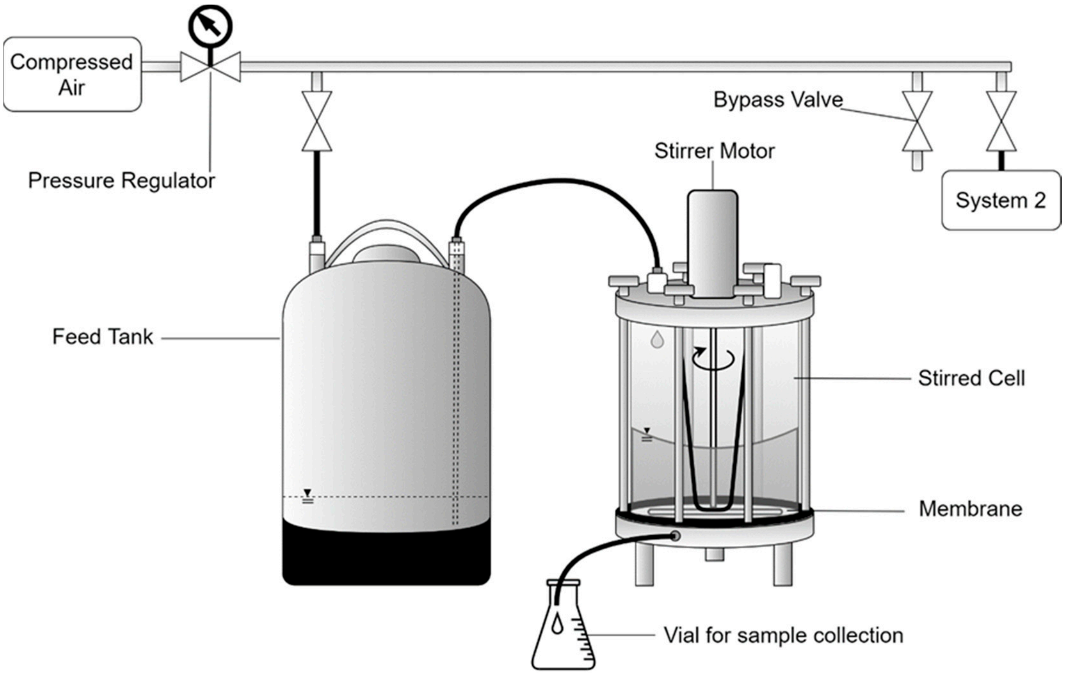

Figure S1. Stirred cell dead end membrane filtration system

2. Ion retention

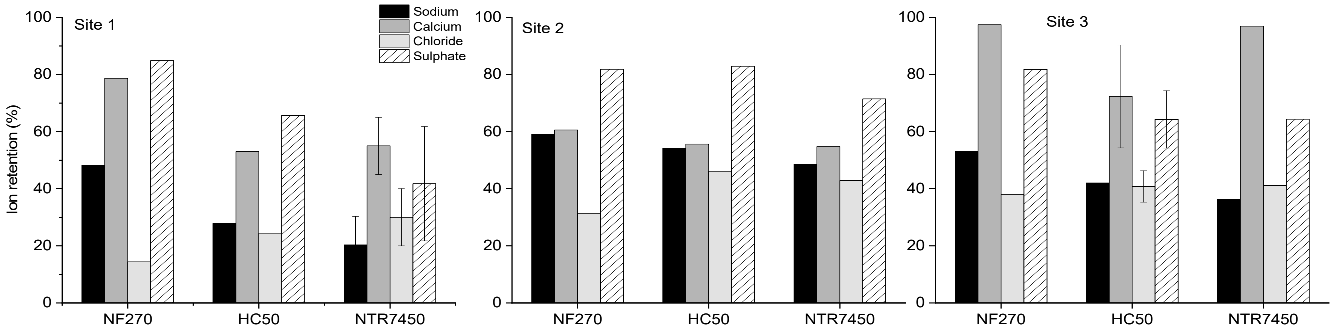

Figure S2. Ion retention of MF permeate at 50% recovery by NF270, HC50 and NTR7450 membranes from all sampling sites. Pressure: 6.5 bar, stirring rate: 400 rpm, temperature: 25°C.

3. COD and DOC retention by NF

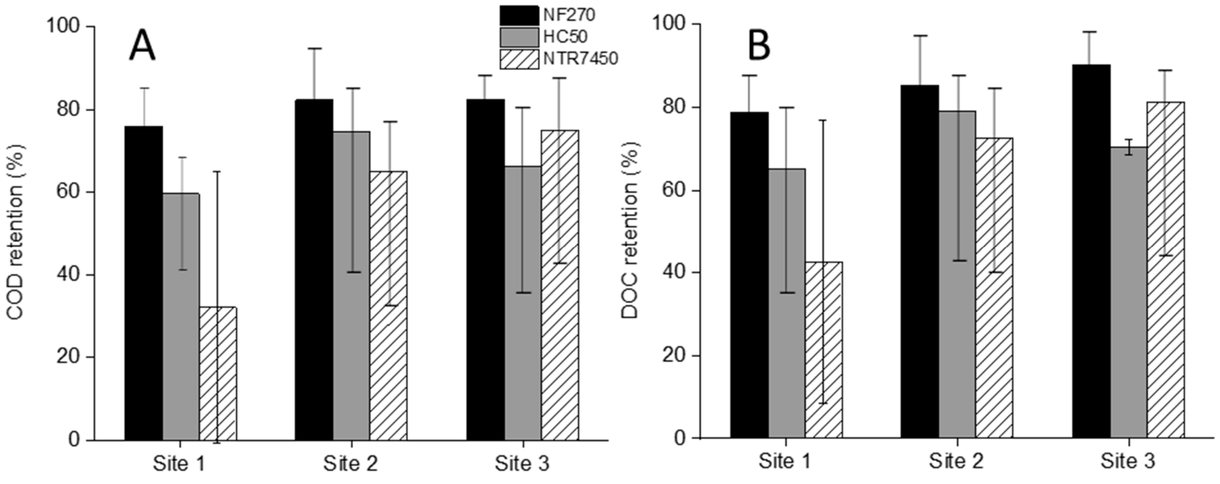

Figure S3. (A) COD and (B) DOC retention of MF permeate at 50% recovery by NF270, HC50 and NTR7450 membranes from all sampling sites. Pressure: 6.5 bar, stirring rate: 400 rpm, temperature: 25°C.

4. Normalized flux during filtration

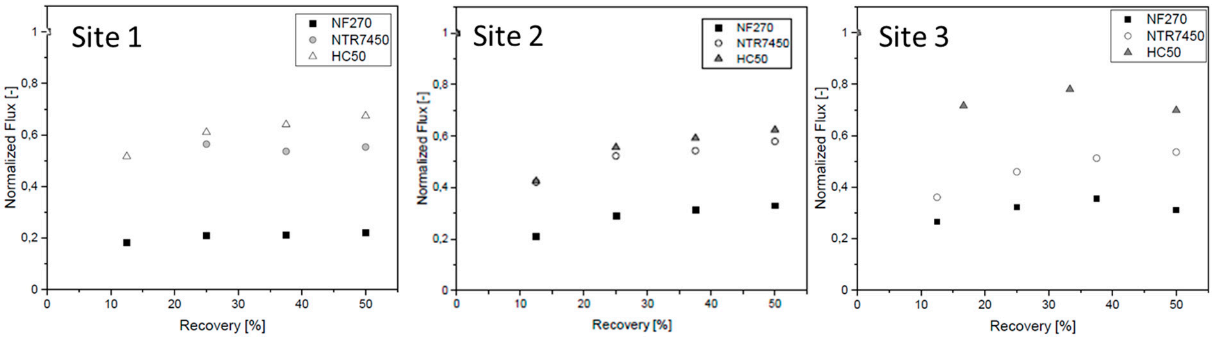

Figure S4. Flux during filtrations of NF270, HC50 and NTR7450 membranes while filtering pig manure from all sampling sites. Pressure: 6.5 bar, stirring rate: 400 rpm, temperature: 25°C.

Normalized flux was calculated by following equation S1, where Jw is pure water flux before MF permeate filtration and Jp is the flux during MF permeate filtration.

$$Normalized\ flux = (Jp/Jw) \qquad \text{(S1)}$$
